# Capillary Sensor for Detection of Amphetamine Precursors in Sewage Water

**DOI:** 10.3390/polym13111846

**Published:** 2021-06-02

**Authors:** Monika Wiśnik-Sawka, Mirosław Maziejuk, Wojciech Fabianowski, Urszula Karpińska, Maciej Szwast, Jerzy Weremczuk

**Affiliations:** 1 Military Institute for Chemistry and Radiometry, Aleja Gen. A. Chruściela “Montera” 105, 00-910 Warsaw, Poland; m.maziejuk@wichir.waw.pl (M.M.); w.fabianowski@wichir.waw.pl (W.F.); 2 Faculty of Chemistry, Warsaw University of Technology, Noakowskiego 3, 00-664 Warsaw, Poland; u.karpinska99@gmail.com; 3 Faculty of Chemical and Process Engineering, Warsaw University of Technology, Warynskiego 1, 00-645 Warsaw Poland; Maciej.Szwast@pw.edu.pl; 4 Faculty of Electronics and Information Technology, Warsaw University of Technology, Nowowiejska 15/19, 00-665 Warsaw, Poland; jweremcz@elka.pw.edu.pl

**Keywords:** BMK detection, sewage water analysis, capillary PP tubing, IMS analysis

## Abstract

This paper deals with the problem of detecting benzyl methyl ketone (BMK), which is a precursor of amphetamine that can be synthesized in home labs. The focus of our work was to identify an improvement for the analysis of sewage introduced into the municipal sewage system. The sensors used to detect BKM in these systems are often clogged and therefore cannot function properly. In this article, a new method of detecting BMK and other chemicals in wastewater is presented. A system containing capillary polypropylene, hydrophobized with polysiloxane coating fibers was prepared. These solutions were used for continuous online measurements by ion mobility spectrometry. The use of pipes with a polysiloxane coating reduces the permeation of water and significantly increases the BMK permeation due to its high solubility in the polymer.

## 1. Introduction

**Figure 1:**
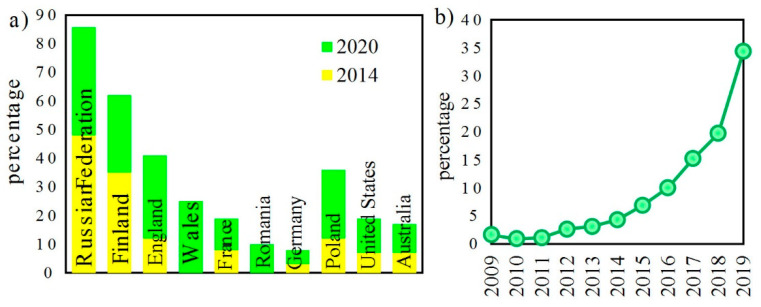
(**a**) Percentage of people in selected countries who use and buy drugs via the darknet in 2014 and 2020; (**b**) percentage of people buying drugs via the darknet for the first time [1].

The last edition of the United Nations report on drugs and crimes 2020 [1] has indicated a rapid increase in illegal drug consumption that occurred between January 2014 and January 2020 (Figure 1).

It can be expected that the situation later in 2020 might be even worse due to the pandemic crisis. Therefore, there is a strong need to localize illegal drug producers. Waste discharged from home drugs laboratories ends up in the sewage system. Such a situation places strong pressure on the detection of substrates and synthesis products in the wastewater. Detection is mainly based on the detection of trace amounts of amphetamine (ATS) and benzyl methyl ketone (BMK) [2].

Unfortunately, the problem of clandestine amphetamine production is especially acute in Europe, where the so-called three “synthetic routes”, the Dutch, the German, and the Polish methods were developed. In their outstanding paper, Frank Hauser and coworkers [3] described the four steps synthesis of amphetamine starting from the BMK precursor α-phenylacetoacetonitrile via Leuckart reaction and the final precipitation of amphetamine sulphate resulting from the addition of strong sulphuric acid to free amphetamine base. There were 27 collected samples of sewage water from the clandestine production and these were subjected to numerous analytical methods including solid phase extraction–gas chromatography–mass spectroscopy (SPE–GC/MS), and capillary electrophoresis method with contactless conductivity detection (CE–CD), giving strong evidence not only for an amphetamine production but also indicating an applied synthetic route. However, these sophisticated analytical tools cannot be applied for fast, online detection working in the real sewage water municipal system.

In the operating sewage water treatment plants [4,5,6,7], there are used advanced sensors for detection of temperature; dissolved oxygen; pH; conductivity; orthophosphate; turbidity; total suspended solids (TSS); ammonium; nitrate; NOx; potassium; chloride; chemical oxygen demand (COD); total organic carbon (TOC); dissolved organic carbon share of TOC (DOC); spectral absorption coefficient (SAC); biochemical oxygen demand (BOD); sludge level; salinity and others; as well as sophisticated detectors for traces of chemicals like ATP or BMK [2,8,9,10]. For example, a specially designed molecular imprinted polymer using a capacitive sensor was designed to detect traces of BMK with a limit of detection (LOD) of 1 µm^2^ in tap water. Nearly all the listed sensors or detectors are in the physical form of a probe, a cylindrical pen-like pipe with a small active part (cap) for molecule detection and measurement. All of them have one serious drawback—working in a real sewage water system, filled with relatively large solid impurities like paper, pampers are easily covered, shadowed with clogging solids, and finally stop to answer to the surrounding chemicals. Probes or detectors operating in the actual sewage system should be resistant to filling with solid waste and protected against direct adhesion to all contaminants. The design must allow direct online measurement or sampling for periodic laboratory testing. We propose a solution to the analysis in a highly contaminated sewage water system with a collection of samples via hydrophobized polypropylene membrane capillaries for ion mobility spectrometer (IMS) analysis or indirect analysis of collected samples in the lab with other techniques such as gas chromatography with a mass spectrometer (GC/MS).

Monitoring of hazardous substances in the environment is a topic that is widely discussed and researched on many levels. One of the primary techniques used to perform screening checks is ion mobility spectrometry (IMS). These detectors are used in portable systems for the analysis of dangerous and psychoactive substances. IMS detectors have more application in laboratory works [11,12,13,14].

Due to the fact that only gas can be fed into the analysis in the IMS detector, appropriate sample introduction systems (SIS) are being developed. One of the solutions used is the use of polymer membranes. The use of such an SIS has been described in over 200 published research papers.

In most of the available studies, the study of systems with the use of a polymer membrane is used in flow systems [15]. In the case of Borsdorf’s group, they used membrane extraction. This technique is highly selective due to the fact that the tert-butyl methyl ether analyzed in this case easily dissolves in a polymer membrane made of polysiloxane, while the membrane is an effective water barrier.

Due to the difficult transport of water through polymer membranes, especially those covered with polysiloxane, they found an application in the constructed systems, allowing for direct collection of an analyte from an aqueous sample and its introduction into a detector [16,17,18]. In these methods, tests are carried out in flow systems. The substance permeating through the membrane is transported by the gas flowing through the inside of the tube and introduced to the detector. Currently, these were performed in the flow of gas through a tube. In the research carried out in this work, the sample is sucked, which creates a negative pressure and accelerates the penetration of the analyte into the tube.

## 2. Materials and Methods

Polypropylene (PP) capillary tubing was supplied by Polymemtech, Poland. Polysiloxane—Sylgard 184 (Dow Corning) was supplied by Milar Biesterfeld Poland. Polastosil M-500; M-2000 were supplied by Silikony Polskie Ltd. (Nowa Sarzyna, Poland). Hexane and chloroform pure for analysis were supplied by Linegal Ltd. Poland. Instrumental analysis (scanning electron microscopy (SEM), water vapor permeation, IMS analysis) were performed at the Warsaw University of Technology and Military Institute of Chemistry and Radiometry (Warsaw, Poland).

Capillary PP membrane tubing [19,20], widely used in water treatment and purification, desalination, and membrane distillation, is usually hollow fibers with a diameter up to 3 mm extruded and formed in the thermal induced phase separation (TIPS) process or dry stretch process (DSP). We have used PP hollow-fiber membrane capillaries produced in the TIPS process [21] at the Warsaw University of Technology. This production process requires the preparation of a creative membrane solution at a temperature of nearly 200 °C. The solution consists of PP granules, a solvent, in this case, soybean oil, and a special agent, castor oil, that makes pores. The homogeneous solution is formed into a capillary shape in a device called a spinneret, and then the formed membrane is cooled in water to coagulate. The solidified capillary is wound on the winding wheel and then cut into the required lengths. The residues of the oils used in the production must then be removed from the membrane by an extraction process. The membranes produced in this process are characterized by a relatively narrow pore diameter distribution, where the average pore diameter is approximately 0.3 µm and surface porosity is approximately 70%. This makes these membranes particularly useful in a microfiltration process but can also serve as support membranes for other coatings, as in the present study.

**Figure 2:**
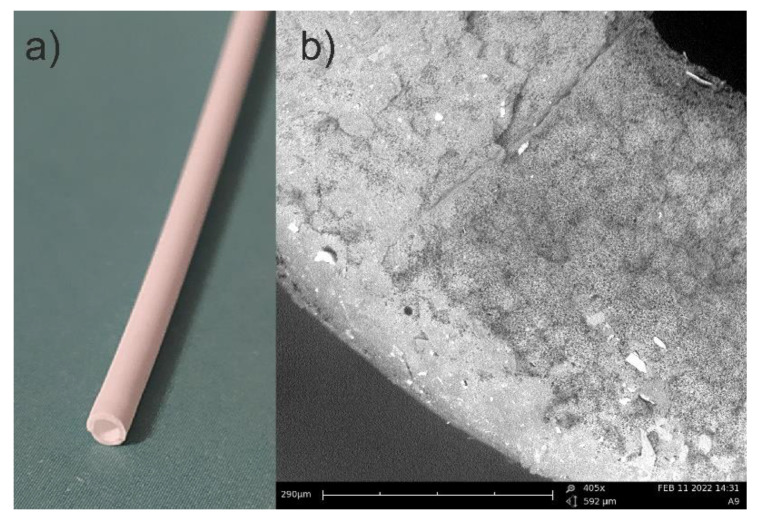
Picture of PP capillary hollow fiber: (**a**) photo; (**b**) SEM picture (note some openings with a diameter of 0.2–0.3 µm).

In Figure 2a, a photo, and in Figure 2b, a picture from the scanning electron microscopy (SEM) are presented. The SEM photo has been taken using PhenomPro device. A special holder for non-conductive samples has been used. This kind of holder allows for testing samples without coating them.

**Figure 3:**
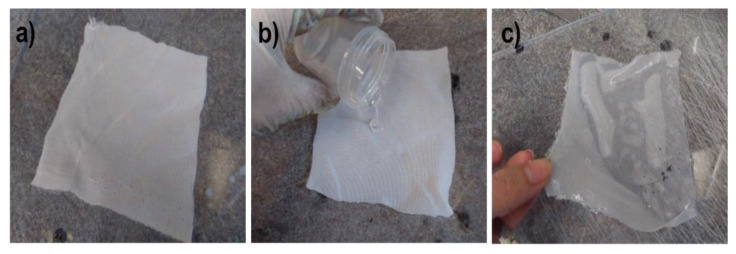
Preparation of PSi membranes for water vapor permeation studies. (**a**) Cotton squares 12 cm × 12 cm; (**b**) pouring PSi onto cotton fabric on a leveled glass plate; (**c**) samples ready for water vapor permeation studies.

The main advantages of capillary PP tubing are related to their low length density (around 1.1 g/m), small diameter of 2.25 mm, and the most important being their high elasticity, allowing them to act in the highly contaminated water sewage as amoeba or protozoa flagella. Nothing adheres to these artificial “flagella” and simultaneously, chemical compounds soluble in water can diffuse into the PP capillary hollow fiber. To limit the ingress of moisture into the tube, the outer surface is covered with a thin hydrophobic layer of polysiloxane (PSi). First, the hydrophobicity of the three used PSi was tested. A simple lab method for PSi membranes reinforced with cotton fabric is presented in Figure 3.

**Figure 4:**
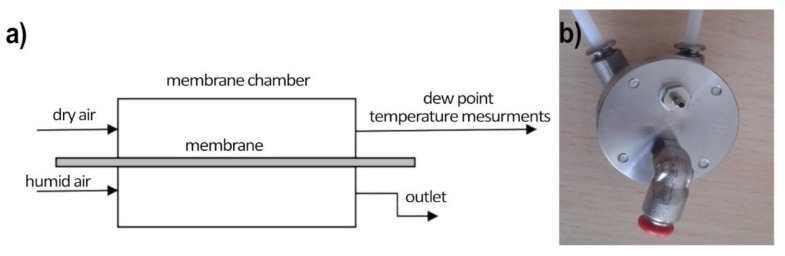
Measurement setup of water vapor permeation across membranes. (**a**) The scheme of the setup; (**b**) top view of the measurement chamber (wet side on the top, dry side on the bottom).

Water vapor permeation studies were performed in the two-stream continuous flow setup: the measurement stand consists of a measurement chamber (made with two sections: wet and dry) with a tested polymer membrane separating the chamber in the middle, the dry air generator (the two-stream reference humidity generator), a humid air generator (water-saturated air at room temperature), and the reference dew point hygrometer (S8000 RS Precision Chilled Mirror Hygrometer), as shown on Figure 4. From the humid air stream, water vapor permeates through the measured membrane which increases the humidity of the dry air stream. This increase is proportional to the membrane water permeability and is registered by the hygrometer.

The measuring chamber is made of stainless steel. Both parts of the chamber were sealed with O-rings to prevent air from getting between the walls of the chamber and the measuring diaphragm. The sealing quality is checked by pressure drop control in time for closed gas installation.

During the measurements, the chamber was supplied with dry and moist air from a humidity generator (described above). Both flows were approximately equal to V = 0.7 L/min. The dew point temperature hygrometer (TDP) was placed on a dry track after the measuring chamber. The tests were carried out on two samples of PSi membranes and a polyethylene membrane at a temperature of 22 °C. The results (averaged from results recorded at the time of 5 min) for TDP and relative humidity (RH) are shown in Table 1.

Similar results were obtained when in place of the PSi membrane separating measuring chamber a thin polyethylene membrane film was used to check the setup and the thin metal membrane was also applied. No water vapor passing was observed.

The obtained results prove that the designed membrane has a good enough separation ability to prevent the diffusion of water vapor.

**Figure 5:**
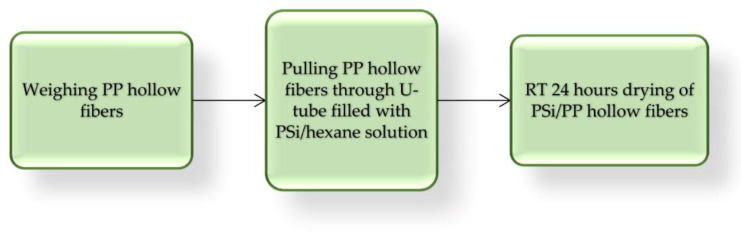
Block diagram of PSi coating PP tubing.

Next, capillary PP hollow fibers 1 m long and 2.26 mm diameter were coated with three PSi by simple pulling, repeated one, two, or three times in U-tube filled with 20 g 10% *w*/*w* hexane PSi solution. This simple lab procedure is presented in Figure 5 and some properties of obtained PP/PSi tubing are listed in Table 2.

For the final analysis of BMK traces in water, we have chosen PP capillaries coated with Sylgard 184, based on our experience with these encapsulating polymers and material for the IMS membranes, which showed superior properties mostly due to its very low content of ionic impurities and extremely low water vapor permeability [22].

## 3. Results

Measurements of BMK in water (BMK concentration 4.04 μg/mL) were performed with a drift tube ion mobility spectrometer (DT IMS) [23], designed and produced in the Military Institute of Chemistry and Radiometry, Poland. DT IMS has its own radioisotope 63Ni source and consists of two regions—ionization region and drift region, separated with a dosage screen. Obtained spectrum is composed of ionized carrier gas particles (so-called reaction ions, RIP) and ionized sample particles (MH^+^). The last ones can be hydrated and can exist as monomeric ions MH^+^(H_2_O)_n_ and dimeric ions (M_2_H^+^(H_2_O)_n_. In the quantitative analysis, both the decay of reactive ion peaks and the raise of sample ion peaks were measured.

**Figure 6:**
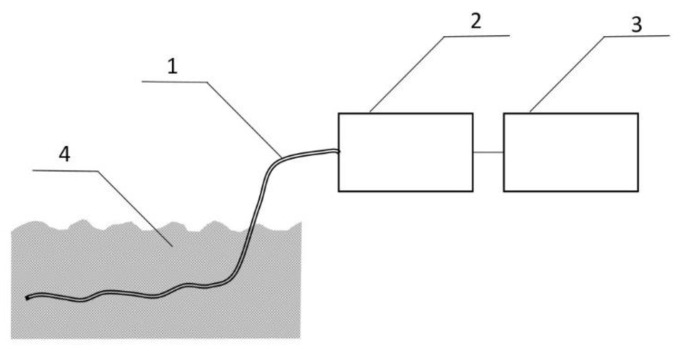
Capillary PP/PSi tubing (1) with one end welded is immersed in water BMK solution mimicking sewage drainage (4). The gas sample is collected with a small air pump (2) connected to the DT IMS analyzer (3).

**Figure 7:**
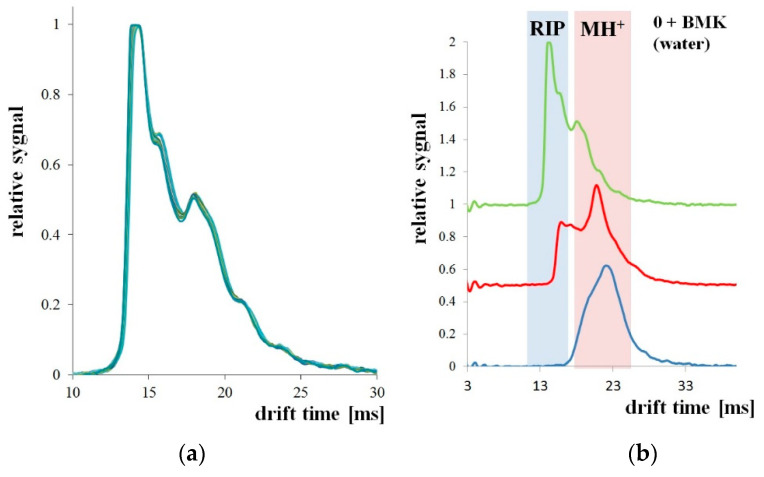
Microporous PP tubing immersed in BMK solution: (**a**) for 30 min; (**b**) for 3 h (1 h).

The measurement system shown in Figure 6 was used to detect BMK in water. The tests were carried out for three types of PP pipes: without PSi coating and with two and three PSi layers. The first attempts were made with a tube without PSi coating. For uncoated PP tubes, the RIP and samples peaks were unchanged for the 30 minutes of the experiment (Figure 7a). After 3 h of the experiment, both the decay of the RIP signal and increase of MH^+^ signal originating from the presence of BMK was observed as shown in Figure 7b (signal at 23 ms drift time).

**Figure 8:**
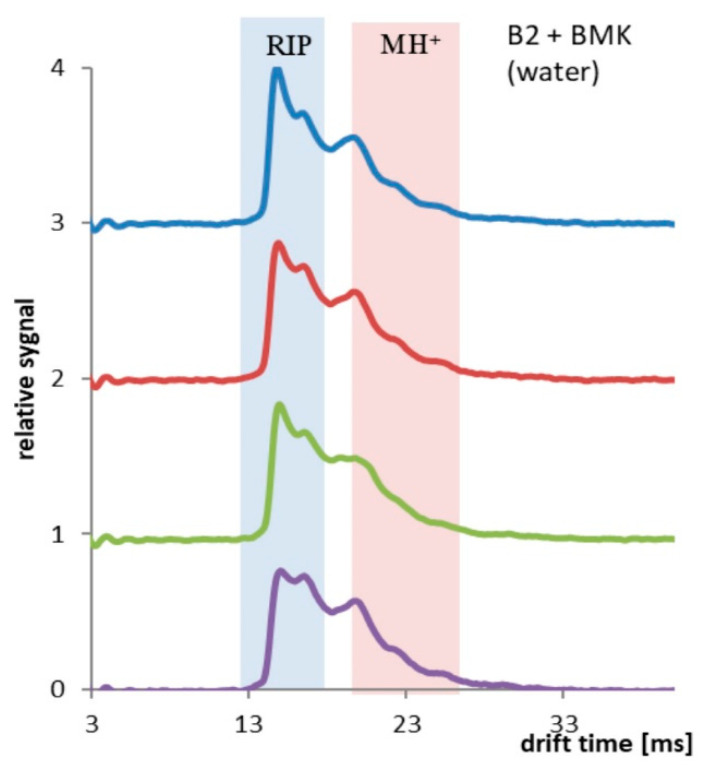
PP tubing coated two times with PSi, spectra collected every 10 min, total time 30 min.

Different results were obtained for the PP tubing coated two times with PSi (Sylgard 184). Hydrophobic coating reduced the permeability of water and increased the solubility and permeation of BMK molecules. In Figure 8, results collected every 10 min (up to 30 min of the experiment) are presented. Decay of RIP signal and increase of MH^+^ signal was observed.

**Figure 9:**
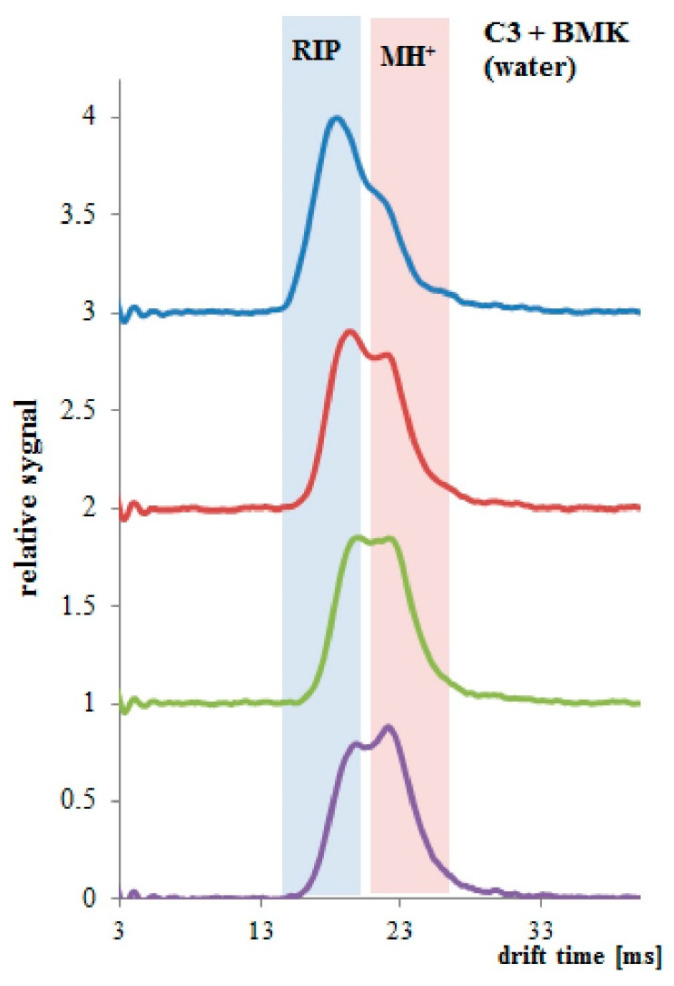
PP tubing coated three times with PSi, spectra collected every 1 min, total time 3 min.

When PP tubing coated three times with PSi was immersed in the BMK water solution, a change in both RIP and MH^+^ signals was observed. Only after 1 min of the experiment, the decay in the RIP signal and raise in the sample signal at the drift time of 23 ms was observed (Figure 9). After 4 min of the experiment, PP/PSi tubing was saturated with the BMK.

As expected, the tube with three PSi layers was the best performer. This was due to the possibility of a significant amount of BMK dissolving in the hydrophobic PSi layer. The slender PP tube was the least effective, in which the penetration into the inside of the tube took place only through the channels located in the material, and not, as in the case of PSi layers, by means of dissolution and diffusion. The use of a highly hydrophobic layer limited the introduction of water to the detector, which allowed the use of this solution for direct sampling from an aqueous solution, without the need to extract the analyte.

IMS analysis is characterized by both high sensitivity and the shortest time of analysis of 60 s. However, the application of IMS analysis for the detection of some drug precursors present in the sewage water system suffered from the easiness of sensor probe contamination and clogging. We have proposed a simple solution using as a probe microporous PP tubing coated with a thin PSi layer. Within 60 s of the experiment, the presence of BMK at a concentration equal to 4 ppm was easily detected. 

In most of the available studies, the analysis of aqueous solutions is carried out in flow systems. This necessitates the performance of specialized sample introduction systems. The method proposed by us solves the situation of collecting samples directly from the flowing sewage, which only contributes to the need to directly connect the sorption tube to the IMS detector. Collection of the sample and its introduction to the detector is performed with the use of a suction pump. Due to the simple structure of the system, it can be used in the construction of modules placed in the sewage system, allowing for continuous monitoring of the discharged sewage waste.

## 4. Patents

Patent application for this effective capillary PP/PSi probe sensor was filled.

## Figures and Tables

**Table 1:** The value of humidity at the inputs and outputs from the measurement system.

Sample	Dry Air Inlet	Humid Air Inlet	Dry Air Outlet
**membrane B3**	**TDP** = −45.5 °C **RH** = 0.26%	**TDP** = 19 °C **RH** = 83.1%	**TDP** = −40.8 °C **RH** = 0.44
**membrane B4**	**TDP** = −46 °C **RH** = 0.24%	**TDP** = 19 °C **RH** = 83.1%	**TDP** = −41.2 °C **RH** = 0.42%
**PET membrane**	**TDP** = −45.5 °C **RH** = 0.26%	**TDP** = 19 °C **RH** = 83.1%	**TDP** = −44.9 °C **RH** = 0.26%

**Table 2:** PP tubing coated with PSi layer.

Sample	PSi	Mass before Coating	Mass after Coating	% Mass Change	Calculated PSi Thickness ^x^	Remarks
		g	g		µm	
0	none	1.331				uncoated
A1	Sylgard 184	1.223	1.507	23	39	1 × coated
A2	Sylgard 184	1.235	1.618	31	52	2 × coated
A3	Sylgard 184	1.281	1.666	30	51	3 × coated
B1	Polastosil M-500	1.280	1.512	18	31	1 × coated
B2	Polastosil M-500	1.220	1.454	19	32	2 × coated
B3	Polastosil M-500	1.252	1.483	19	32	3 × coated
C1	Polastosil M-2000	1.209	1.527	26	44	1 × coated
C2	Polastosil M-2000	1.242	1.548	25	42	2 × coated
C3	Polastosil M-2000	1.217	1.526	25	42	3 × coated

^x^—assuming PSi13 density 1.03 gcm^−3^.
